# The role of PoCUS in the assessment of COVID-19 patients

**DOI:** 10.1007/s40477-021-00586-8

**Published:** 2021-04-19

**Authors:** John Karp, Karina Burke, Sarah-Marie Daubaras, Cian McDermott

**Affiliations:** 1 School of Medicine, Royal College of Surgeons in Ireland, Dublin, Ireland; 2 Emergency Department, St James Hospital, Dublin, Ireland; 3 Emergency Department and Emergency Ultrasound Education, Mater University Hospital, Dublin, Ireland

**Keywords:** PoCUS, Ultrasound, COVID-19, Coronavirus

## Abstract

The Coronavirus disease 19 (COVID-19) pandemic has increased the burden of stress on the global healthcare system in 2020. Point of care ultrasound (PoCUS) is used effectively in the management of pulmonary, cardiac and vascular pathologies. POCUS is the use of traditional ultrasound imaging techniques in a focused binary manner to answer a specific set of clinical questions. This is an imaging technique that delivers no radiation, is inexpensive, ultraportable and provides results instantaneously to the physician operator at the bedside. In regard to the pandemic, PoCUS has played a significant adjunctive role in the diagnosis and management of co-morbidities associated with COVID-19. PoCUS also offers an alternative method to image obstetric patients and the pediatric population safely in accordance with the ALARA principle. Finally, there have been numerous PoCUS protocols describing the effective use of this technology during the COVID-19 pandemic.

## Introduction

COVID-19 is a novel virus from the coronavirus family. It has a mean incubation period of 4–5 days however, [1, 2] COVID-19 spreads via respiratory droplets from person-to-person and causes an acute respiratory illness [3]. While the information on the pathogenesis and pathology of the disease is quickly evolving and vast with large variations on the severity of disease, the major symptoms include fever, fatigue, dry cough, dyspnea and anosmia [4]. Since notification of the first instances of a cluster of pneumonias with an unknown cause in Wuhan, China on the 31st of December 2019, COVID-19 has spread quickly throughout 216 countries globally [4]. Declared a pandemic by the WHO on March 11th, 2020, the disease has led to 35 million infections and 1 million deaths [5]. The rise of COVID-19 globally has placed significant pressure on healthcare and its resources.

The current gold standard diagnostic method is reverse transcription-PCR (RT-PCR) testing of a nasopharyngeal swab [6, 7]. Alternatively, chest X-ray (CXR) and chest computed tomography (CT) are commonly used imaging modalities. A CXR is less sensitive than a chest CT and they both subject the patient to varying degrees of radiation [8, 9]. PoCUS refers to the act of using ultrasound during a standard clinical assessment as an adjuvant diagnostic tool. Ultrasonography has proven to be an alternative solution in offering a safe and quick first-line bedside diagnostic exam for COVID-19 lung, cardiac and venous thromboembolic manifestations [10–12]. In this review, the implications of PoCUS in the management of COVID-19 patients will be explored.

## Methods

To complete this literary review, articles from PubMed.org database were reviewed for eligibility. No language, date or age restrictions were applied. A search string was designed using the following terms: COVID-19, coronavirus, point of care ultrasound, and PoCUS with the following filters applied: clinical trial, meta-analysis, randomized controlled trial, review, systematic review. Papers deemed eligible based on abstract review were accessed as full-length articles for analysis.

### PoCUS advantages and limitations

Nanotechnological advancements in technology have enabled ultrasound to become an affordable and portable imaging modality. Whether it is a cart-based ultrasound machine or a handheld device, a scan can be conducted at the bedside quickly and affordably while providing physicians with results in real time [13, 14]. In regard to COVID-19, PoCUS offers a non-invasive option to screen and trend patients safely. Disinfection protocols include sheathing and disinfection of the ultrasound transducer [10]. Although an ultrasound device may act as a fomite for COVID-19, following a simple checklist can assist physicians in the disinfection process to safely reduce contamination (Table 1) [11, 15–18].

**Table 1:** Modified PoCUS machine disinfection checklist: COVID-19 point-of-view

Pre-Scan	Minimize the amount of additional equipment on the ultrasound cart and if possible designate certain devices as COVID-19-specific
	If possible, cover the machine and probe in a protective plastic sheath
	Use single-use gel packets rather than a reusable bottle
Post-Scan	Inspect the probe and thoroughly sanitize the machine/reusable plastic sheath using approved-disinfectant wipes while wearing personal protective equipment (PPE)^a^
	Remove the machine from the patient’s room followed by doffing PPE
	Reinspect the machine; re-apply new gloves and disinfect the machine once again

^a^ Disinfecting hand-held PoCUS device can be less strenuous with the use of smaller disposable plastic sheaths

Additionally, the portability of PoCUS reduces the risk of transmission to staff and equipment exposure, which in turn conserves valuable personal protective equipment (PPE) and supplies [11]. Rather than transporting a patient through the hospital to the radiology department, a dedicated ultrasound machine in a COVID-19 unit may be brought to the patient’s bedside. A single physician can interact with the patient minimizing staff exposure while performing an exam.

In comparison to CT imaging, PoCUS is a non-ionizing imaging modality which offers comparable sensitivity and accuracy in the diagnosis of COVID-19 lung involvement [19]. In comparison to a portable X-ray which correlated to CT lung findings by 62%, PoCUS paralleled CT lung findings of COVID-19 by 87% [20]. Another study concluded that while comparing ultrasound to CT images of alveolar-interstitial patterns in the same COVID-19 patients, PoCUS had a sensitivity of 60% compared to only 39% in CT images [21]. Additionally, PoCUS is extremely beneficial in monitoring the progression of cardiac and venous abnormalities associated with COVID-19 [11].

Although PoCUS is a useful tool, it does carry limitations. To acquire clinically useful images, the operator must have sufficient experience using an ultrasound machine [11, 13]. Patients with an elevated body habitus or compromised status may impair the operator’s ability to obtain a clear visual [22]. In addition, PoCUS images may be difficult to interpret, which may lead to missed findings or require further investigations using alternative imaging modalities [11].

### PoCUS for COVID-19 lung involvement

The organ system most commonly involved by the coronavirus is the respiratory tract. It can manifest as a mild viral pneumonia or acute respiratory distress syndrome [23, 24]. Lung ultrasound (LUS) has proven to be an invaluable first-line diagnostic tool when it comes to COVID-19 patients. It has an overall diagnostic sensitivity and specificity of 90.2% (67.6–99.3%) and 88.8% (81.8–94.4%) in COVID-19 pneumonia when compared to CXR, CT and clinical exam [25]. Additionally, while COVID-19 has a preference to affect the posterior-basal lung zones, ultrasound provides effective visualization of the lung’s peripheries [10, 26–30]. LUS is not capable of visualising central lung lesions—these are best seen on cross-sectional CT imaging.

Common pathologies visualized using LUS during COVID-19 are multifocal B lines, sub-pleural/lobar consolidations with decreased doppler signal, bilateral alveolar-interstitial pattern, pleural irregularities, air bronchograms and less commonly pleural effusions [9, 11, 18, 30–33]. B-lines are defined as reverberation artifacts that are represented as smooth vertical lines that extend from the pleural line to the far field while obliterating A-lines [34, 35]. Particularly in COVID-19, B-lines are commonly visualized in the posterior-lateral lung zones in the early phases of the disease [33]. In addition to pleuropathies, consolidations are pathological and can be identified peripherally and inferior to or disrupting the pleural line [9, 18, 30, 31]. Air bronchograms are visualized as bright opacities distal to a consolidation when the ultrasound wave reflects off of an air-filled bronchus [9, 18, 31]. As previously stated, pleural effusions are uncommon in COVID-19 but can be seen clearly as anechoic regions found cephalad to the diaphragm [9, 18, 30, 31].

### PoCUS for COVID-19 cardiac involvement

PoCUS has played a pivotal role in monitoring patients’ cardiac function, especially those with pre-existing cardiovascular co-morbidities [10, 12]. A point-of-care echocardiogram may be useful to evaluate patient status when they become hemodynamically unstable or have elevated cardiac biomarkers [12]. Cardiac involvement in COVID-19 presents in a large proportion of patients and as a multitude of pathologies. Cardiac involvement of the disease is very common amongst patients and the process can be either via primary viral infection or secondary activation of the immune cascade [14, 23]. These processes directly affect cardiac function from resultant myocarditis and pulmonary embolisms (PE) [17, 23].

PoCUS has high accuracy when used to evaluate left and right ventricular function, valvular dysfunction, pericardial effusion and to calculate stroke volume [10, 36]. In monitoring PE occurrence, PoCUS has been used to assess right ventricular function and resulting pulmonary hypertension. The ability to identify these findings using cardiac PoCUS provides information for whether or not additional management is required [37].

### PoCUS for COVID-19 vascular involvement

Fluid status changes and vascular abnormalities are commonly associated with COVID-19. It has been suggested that inflammation caused by COVID-19 may increase vascular permeability, thus reducing intravascular volume [38]. The hepatic, portal and intra-renal veins can be visualized using PoCUS to accurately assess fluid status in hospitalized COVID-19 patients [36]. If PoCUS is conducted serially, it is extremely beneficial in monitoring the effect of fluid administration in hemodynamically unstable patients [14].

Similarly, to other critically ill patients, COVID-19 has been associated with an increased risk for thromboembolic events [11]. A two-point compression PoCUS ultrasound examination may be utilized to assess for deep vein thrombosis (DVT) in the femoral and popliteal veins [11, 36]. Given the difficulties in diagnosing a PE with superimposed lung involvement using CT pulmonary angiography, venous doppler studies are essential in guiding appropriate management [14].

Ultrasound-guided venous access provides physicians with an abundance of advantages. PoCUS can be utilized to assess for vessel thrombi prior to intravenous puncture, it may also help distinguish a vein from the artery [12]. It can also assess lung sliding pre- and post-procedural during central line placement to diagnose iatrogenic pneumothorax [12]. Particularly in COVID-19 patients, these parameters are very important to account for to prevent precipitating a thromboembolic event or worsening of concomitant lung involvement.

### PoCUS for COVID-19 in pregnancy

PoCUS is particularly useful in pregnant women and monitoring disease progression of COVID-19 [39–42]. Accessibility at the bedside and elimination of radiation exposure enables ultrasound to be used to serially track COVID-19 manifestations while pregnant [40]. Although CT imaging is used to diagnose and monitor pulmonary disease progression in many COVID-19 patients, the downsides of radiation exposure outweigh the benefits. In theory, reducing the amount of radiation used would be the solution, however, CT with reduced radiation levels has been found to be insensitive in the early and/or mild COVID-19 cases [43].LUS is proven to correlate with CT images and is therefore feasible during pregnancy [3, 44].

In addition, obstetricians and gynecologists may be adequately trained to perform a fetal ultrasound followed by a LUS examination in pregnant patients with a suspected or known COVID-19 diagnosis [45, 46]. This minimizes the need for various ultrasound operators and equipment, reducing the exposure to potential infection. Moreover, the severity of the disease seen on LUS may influence treatment decisions and subsequently the outcome of the pregnancy [43].

### PoCUS for COVID-19 in neonates and children

Ultrasonography is a safe and effective tool to monitor neonatal lung and cardiac diseases. Its neonatal application in COVID-19 has been used to assess the progression of lung involvement [47–50]. Interestingly, some neonates with COVID-19 were found to have signs of lung involvement on LUS, that did not coincide with any respiratory decompensation, commonly seen in adults [51]. Similarly to adults, B-lines, pleural irregularities, and consolidations were noted in neonates [51].

Although much of the discussion around COVID-19 and ultrasound refers to its use in adults, it is also a practical tool when it comes to assessing infection in children. A majority of children are asymptomatic, yet those who present with symptoms commonly have a fever, cough or both [52]. It has been suggested that in early disease, not all children display characteristic COVID-19 LUS findings [53]. However, when they do, ultrasound findings include pleural irregularities, sub-pleural consolidations and B lines [54]. Therefore, in addition to the use of LUS in other viral pneumonias, there is compelling evidence suggesting LUS should be used at the bedside in children [54].

Less frequently, children have been reported to develop multi-system inflammatory syndrome (MIS); a syndrome which closely mimics Kawasaki disease [55]. This can present as a rash, bilateral conjunctivitis, myocardial dysfunction, shock, coagulopathy or acute gastrointestinal distress [55]. A large majority of these children with MIS present with cardiac manifestations including arrhythmias, ventricular dysfunction and coronary dilatation, primarily caused by focal necrosis and fibrosis of the myocardium [33]. Furthermore, to prevent the overuse of CT on children, ultrasound may be a useful modality in the evaluation of cardiac dysfunction, fluid status and vascular abnormalities in the COVID-19 pediatric population [54–56].

### PoCUS scanning protocol options in COVID-19

As a result of COVID-19, numerous PoCUS scanning protocols have been introduced to best assess patient status. (Table 2) Each protocol was designed to quickly assess COVID-19 patients in the emergency department or the intensive care unit. This not only minimizes exposure to potential respiratory droplets but also gives the physicians a more well-rounded clinical picture of the patient’s current condition.

**Table 2:** Summaries of PoCUS Protocols

	Date Introduced for COVID19	Authors	Pulmonary assessment	Lung zones	Cardiac assessment	Vascular assessment	Details (Position, transducer, system examined, time required)		Population studied
End SBT Protocol [59]	March 12th, 2020	Peng et al.	✓	12				Originally for patients with postextubation distress Upper and lower posterior zones Upper and lower axillary zones Upper and lower anterior zones	20 adult patients at Xiangya Hospital [60]
LUSCOVID Protocol [33]	March 30th, 2020	Soldati et al.	✓	14			10 s per zone Linear/convex transducer Basal, middle and upper paravertebral line Basal and upper midaxillary line Basal and upper midclavicular line		1462 COVID-19 patients across 20 USA/European hospitals [25] 1 pregnant woman with COVID-19 at a tertiary hospital in Rome [40 10 children with COVID-19 at 2 tertiary hospitals in Rome [54] 88 adult COVID-19 patients from 3 Italian hospitals [28]
ASE Protocol [11]	April 15th, 2020	Johri et al.	✓	8–10	✓	✓	Assess ventricles, pericardium and valves Perform an 8–12 zone lung scan Assess the jugular venous pressure (JVP), subcostal inferior vena cava (IVC) and leg veins		77 COVID-19 patients in the Adan General Hospital ICU [28]
DLETE Protocol [61]	April 18th, 2020	Fox et al.	✓	8	✓	✓	10-min examination Linear/phased array transducer Perform a focused ECHO Perform an 8-zone lung scan Assess the IVC and leg veins for DVTs		–
CLUE Protocol [6]	May 9th, 2020	Manivel et al.	✓	12			Upright position Linear/convex transducer Upper and lower posterior zones Upper and lower lateral zones Upper and lower anterior zones		–
BLUE^a^ Protocol [57]	May 15th, 2020	Karagöz et al.	✓	12			Upright position Upper and lower posterior zones Upper and lower axillary zones Upper and lower anterior zones		20 adult patients at Xi’an Chest Hospital [58]
Six Zone Protocol [30]	June 12th, 2020	Antúnez-Montes et al.	✓	6			Upright/prone position Linear/curvilinear transducer Bilateral posterior, lateral superior, and lateral inferior zones		38 COVID-19 patients in Tongji Hospital, Wuhan [29]

^a^The BLUE protocol was initially introduced by Lichtenstein et al., however, was first outlined for its use in COVID-19 by Sultan et al. [57, 65]

**Fig. 1.**
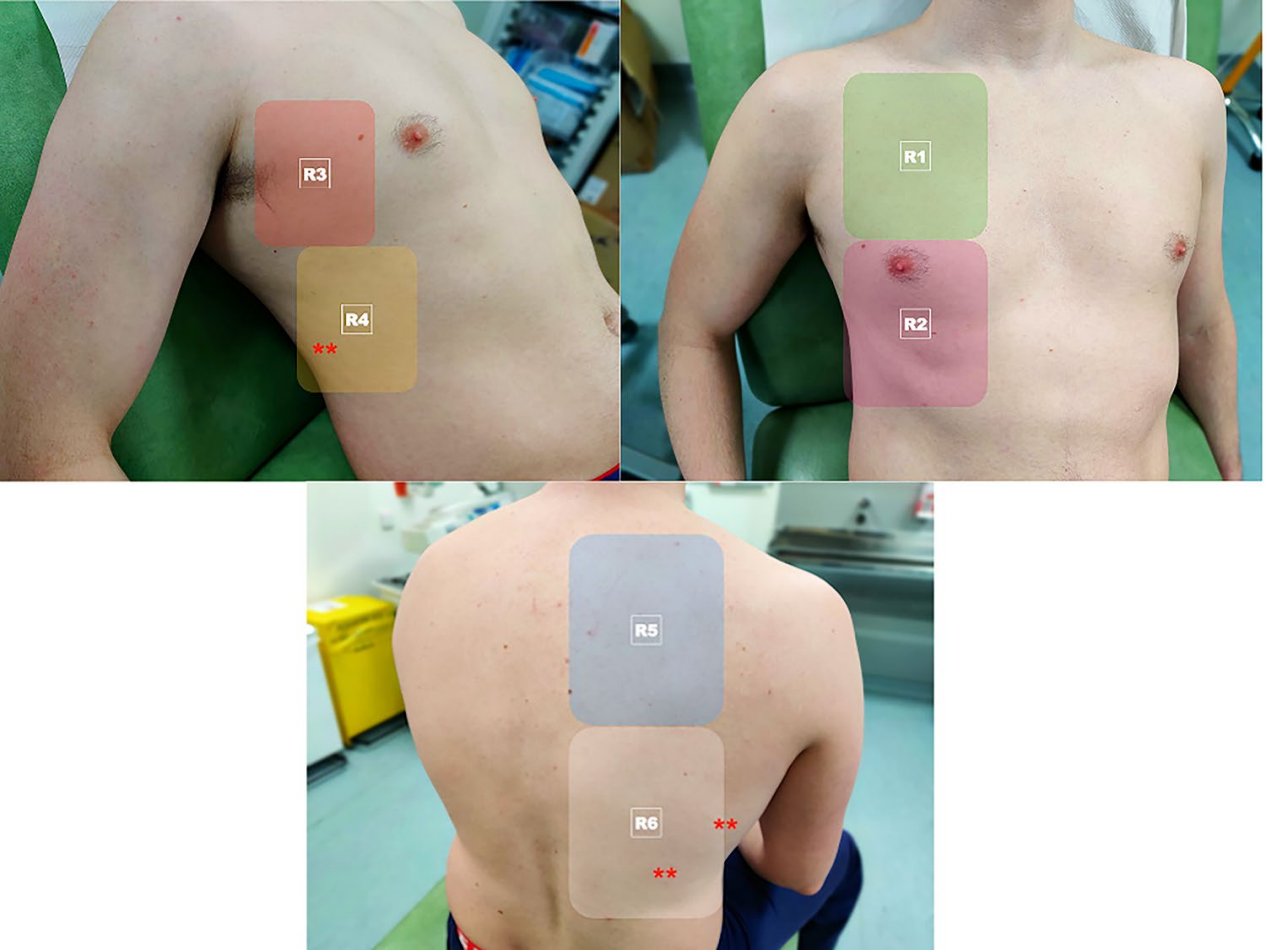
12 zone scanning protocol showing 2 anterior zones, 2 lateral zones and 2 posterior zones. Asterisks (*) indicate the posterobasal areas where COVID19 changes occur most frequently

The ‘under 2-min’ Six Zone LUS Protocol [29, 30] proposes that the operator situates themselves posterior to the patient sitting upright, and three scans are conducted bilaterally: posterior zone of the chest wall, lateral superior and inferior zones of the axilla (Fig. 1). The CLUE Protocol [6], BLUE Protocol [57, 58] and the end spontaneous breathing trial (SBT) Protocol [59, 60] involve a more comprehensive twelve-zone LUS assessment. The CLUE Protocol produces a score of 0–3 per zone, with 3 representing more severe disease. The BLUE Protocol offers insight into what the underlying pulmonary pathology may be depending on the specific LUS findings observed. In regard to COVID-19, the presence of multiple diffuse B-lines indicates pulmonary edema, whereas consolidations indicate pneumonia [57]. The end SBT LUS protocol provides a score of 0 for a normal LUS, 1 or 2 for the presence of B-lines and 3 for consolidation [59]. The LUSCOVID Protocol [25, 28, 33, 39, 54, 61, 62] assesses 14-zones thus it is the most extensive LUS protocol.

The ASE Protocol [11, 63] and DLETE [64] are the most comprehensive and are comprised of cardiac, lung and venous assessment which encompasses the main manifestations of COVID-19.

## Conclusion

PoCUS’ usefulness as an adjunctive tool in the management of COVID-19 is strong. The portability, availability and the real-time nature of ultrasound provides physicians with a safe imaging modality for pulmonary, cardiac and vascular COVID-19 manifestations. With no risk of radiation exposure, ultrasonography is beneficial when used to assess lung involvement in pregnant women. Additionally, ultrasound is practical and effective for use in pediatric COVID-19 patients.
